# Skin photoprotective and antiageing effects of a combination of rosemary (*Rosmarinus officinalis*) and grapefruit (*Citrus paradisi*) polyphenols

**DOI:** 10.3402/fnr.v60.31871

**Published:** 2016-07-01

**Authors:** Vincenzo Nobile, Angela Michelotti, Enza Cestone, Nuria Caturla, Julián Castillo, Obdulio Benavente-García, Almudena Pérez-Sánchez, Vicente Micol

**Affiliations:** 1 Complife Group, Pavia, Italy; 2 Monteloeder S.L., Alicante, Spain; 3 Nutrafur S.A. (Frutarom Group), Murcia, Spain; 4 Universidad Católica San Antonio, Murcia, Spain; 5 Institute of Research Into Aging, University of Murcia, Murcia, Spain; 6 Instituto de Biología Molecular y Celular, Universidad Miguel Hernández (UMH), Alicante, Spain

**Keywords:** plants extracts, *Rosmarinus officinalis*, *Citrus paradisi*, clinical study, photoprotection, antiageing

## Abstract

**Background:**

Plant polyphenols have been found to be effective in preventing ultraviolet radiation (UVR)-induced skin alterations. A dietary approach based of these compounds could be a safe and effective method to provide a continuous adjunctive photoprotection measure. In a previous study, a combination of rosemary (*Rosmarinus officinalis*) and grapefruit (*Citrus paradisi*) extracts has exhibited potential photoprotective effects both in skin cell model and in a human pilot trial.

**Objective:**

We investigated the efficacy of a combination of rosemary (*R. officinalis*) and grapefruit (*C. paradisi*) in decreasing the individual susceptibility to UVR exposure (redness and lipoperoxides) and in improving skin wrinkledness and elasticity.

**Design:**

A randomised, parallel group study was carried out on 90 subjects. Furthermore, a pilot, randomised, crossover study was carried out on five subjects. Female subjects having skin phototype from I to III and showing mild to moderate chrono- or photoageing clinical signs were enrolled in both studies. Skin redness (a* value of CIELab colour space) after UVB exposure to 1 minimal erythemal dose (MED) was assessed in the pilot study, while MED, lipoperoxides (malondialdehyde) skin content, wrinkle depth (image analysis), and skin elasticity (suction and elongation method) were measured in the main study.

**Results:**

Treated subjects showed a decrease of the UVB- and UVA-induced skin alterations (decreased skin redness and lipoperoxides) and an improvement of skin wrinkledness and elasticity. No differences were found between the 100 and 250 mg extracts doses, indicating a *plateau* effect starting from 100 mg extracts dose. Some of the positive effects were noted as short as 2 weeks of product consumption.

**Conclusions:**

The long-term oral intake of Nutroxsun™ can be considered to be a complementary nutrition strategy to avoid the negative effects of sun exposure. The putative mechanism for these effects is most likely to take place through the inhibition of UVR-induced reactive oxygen species and the concomitant inflammatory markers (lipoperoxides and cytokines) together with their direct action on intracellular signalling pathways.

Exposure to solar ultraviolet radiation (UVR) is one of the most important environmental factors affecting skin physiology. Solar UVR reaching the earth's surface can be classified according to wavelength as UVB (290–320 nm) and UVA (320–380 nm) radiation. UVA and UVB radiation ratio reaching earth's surface is 95%:5%, and it is dependent on geo-orbital factors (latitude, season, time) and on environmental factors (ozone layer, cloud thickness, pollutants, UV rays reflection from ground) (1, 2).

Historically, UVB radiation has been considered responsible for early and late consequences of solar UVR exposure. UVB radiation is, in fact, the main cause of the cardinal sign of acute solar UVR exposure: the erythema sign characterising the inflammatory reaction typical of sunburn (3–7). Erythema starts approximately 3–5 h after UVB radiation exposure, reaches its maximum at 12–24 h, and fades over 72 h (8). Skin inflammation due to acute exposure to UVR has been shown to be characterised by the release of neuropeptides, histamine, prostaglandins, serotonin, and oxygen radicals (4–6, 9, 10), and the upregulation of pro-inflammatory cytokines such as interleukin 1 (IL-1), interleukin 6 (IL-6), and tumour necrosis factor alpha (TNF-α) (11–16). Histologically, sunburn is characterised by dyskeratotic and vacuolated keratinocytes (sunburn cells), mild epidermal spongiosis, depletion of Langerhans cells, dermal oedema, endothelial cell enlargement, and later by a neutrophilic dermal infiltrate (17). Accumulated evidence on the effects of prolonged or repetitive UVB exposure during the past two decades has been reported to lead to generalised immunosuppression leading to carcinogenesis (18, 19) through the production/secretion of anti-inflammatory cytokines such as IL-4 and IL-10 (11, 20, 21).

In recent years, an increasing use of artificial sources of UVA radiation both for medical treatment (phototherapy and photochemotherapy) and for aesthetic purposes (solarium) has revealed the harmful role of UVA radiation in the pathophysiology of skin alterations due to sun exposure. UVA radiation penetrates deeper within the skin and is mostly responsible for the generation of reactive oxygen species (ROS) including singlet oxygen (^1^O_2_), and other non-radical and radical ROS, such as hydrogen peroxide (H_2_O_2_) and the superoxide radical (O_2_^•–^) (22–26) and, to a lesser extent than UVB radiation, can also induce DNA damage (27, 28). UVA-induced oxidative stress increases the potential for reactions like the oxidation of lipids and proteins (29). Both UVA and UVB contribute significantly to photoageing.

The protection of the skin from solar exposure is consigned to topical sunscreens. However, topical sunscreens have drawbacks including seasonal application (generally sunscreens are applied only during holidays) and inadequate application methods (e.g. quantity and spreading). Furthermore, the sun protection factor (SPF) provided by sunscreens seems to be overestimated, under testing conditions, when compared to the real-life condition of use (30, 31). Therefore, a dietary approach to photoprotection could be an effective method to provide a continuous adjunctive protection measure, with population-level impact (32).

Several plant extracts have been found to be effective in preventing UV-induced skin alterations. The most important group of compounds includes phenolic acid, flavonoids, and high-molecular-weight polyphenols (33–39). Several studies have shown the flavonoids to act as scavengers of superoxide anions, singlet oxygen, hydroxyl radicals, and lipid peroxyl radicals (37, 40–43). There are also reports of flavonoids inhibiting the activities of many enzymes, including lipoxygenase, cyclooxygenase, monooxygenase, xanthine oxidase, mitochondrial succinate dehydrogenase, NADH oxidase, phospholipase A2, protein kinases, and nuclear transcription factor (NF-κB) (44, 45).

A previous study demonstrated the efficacy of a commercially available mixture of citrus and rosemary extracts (Nutroxsun™, Nutrafur S.A. & Monteloeder S.L., Spain) in skin cell models and on humans. The following was reported: 1) a protective effect on cells viability after UVB radiation, a decrease of UVB-induced intracellular ROS, and prevention of DNA damage in an immortalised human keratinocyte cell line (HaCaT), 2) a decreased chromosomal aberrations in X-irradiated human lymphocytes, and 3) an increase of the minimal erythemal dose (MED) 8 and 12 weeks after the oral daily consumption (250 mg) of the extracts (46). Based on these preliminary data, in the current study we investigated the efficacy of the tested product (100 and 250 mg doses) in decreasing the individual susceptibility to UVR exposure (redness and lipoperoxides [LPO]), in decreasing wrinkle depth, and in improving skin elasticity. To our knowledge, no other studies have investigated the photoprotective and antiageing efficacy of the tested product.

## Methods

### Study design

This was a monocentric, randomised, crossover (short-term study), parallel group (long-term study) study conducted in Italy. The study protocol and the informed consent form were approved by the ‘Independent Ethical Committee for Non-Pharmacological Clinical trials’ during its meeting on 15 December 2014. All subjects provided written informed consent before initiation of any study-related procedures. No changes to treatment regimen or to methods were necessary after study starting.

### Subjects

Eligible subjects were all adult, female subject, having skin phototype from I to III (Fitzpatrick classification) (47) and showing mild to moderate chrono- or photoageing clinical signs. The subjects were of general good health, had no alimentary/eating disorders (i.e. bulimia, psychogenic eating disorders, etc.), and have known history of metabolic syndrome. Exclusion criteria were pregnancy or intention to become pregnant, lactation, food intolerances/allergy, pharmacological treatments known to interfere with the test product or having an effect on metabolism, participation in another similar study, unwillingness or inability to comply with the requirements of the study protocol. The study further excluded subjects using food supplements containing active ingredients that have an influence on skin response to UV rays or on skin ageing. During all the study period, subjects were asked to avoid any UV exposure (artificial UV light or sunlight). The study took place at Farcoderm Srl facilities in San Martino Siccomario (PV), Italy. Farcoderm Srl is an independent testing laboratory for *in vitro* and *in vivo* safety and efficacy assessment of cosmetics, food supplements and medical devices.

### Intervention

The test product was a commercially available mixture of rosemary and citrus extracts (Nutroxsun™, supplied by Monteloeder S.L., Miguel Servet 16, Elche, Alicante, Spain), obtained from dried rosemary (*Rosmarinus officinalis*) leaves and grapefruits (*Citrus paradisi*), respectively. Nutroxsun™ total phenolic standard content is higher than 35 gallic acid equivalents/100 g dry weight (dw) as determined by Folin assay (48), being the total rosemary phenolic content higher than 7% dw and total grapefruit flavones content higher than 20% dw.

**Fig. 1:**
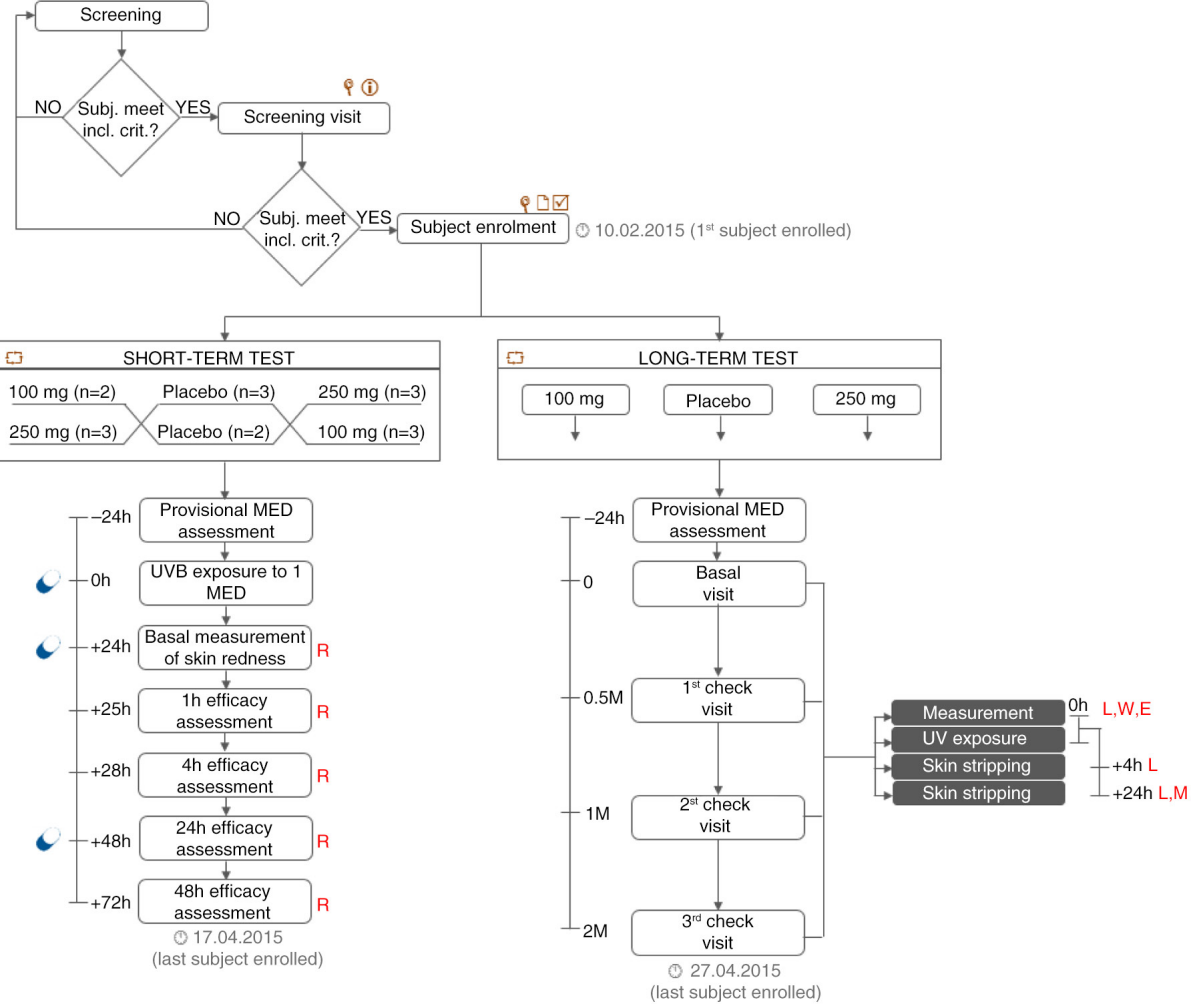
Study flow and schedule of assessments chart. Subjects were first screened in the Farcoderm volunteers database (keywords: Sex = ‘female’, Age = ‘18’, Skin phototype = ‘I < phototype < III’, Skin type: ‘ageing or photoageing’, Testing preferences: ‘food supplements’). Eligible participants were then screened by a board certified dermatologist. During the screening visit, a physical examination was carried out in order to assess the uniformity of the test area (back) and the clinical sign of skin ageing on the face. Subjects meeting the inclusion criteria were then enrolled and randomised to participate in the short- or in the long-term study. Legend: 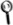, physical examination; 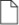, informed consent signature; 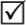, eligibility check; 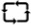, randomisation; PM*, provisional MED measurement (carried out only before study start); M, MED; L, lipoperoxides; W, wrinkle depth; E, skin elasticity; R, skin redness 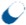 product intake.

Both in the long-term and in the short-term tests, subjects were randomly assigned to receive 100 mg Nutroxsun™, 250 mg Nutroxsun™, or the placebo (100% maltodextrin) product. In the short-term study, subjects received the first dose (100 or 250 mg) of the test product or the placebo product 15–30 min before UVB exposure to 1 MED. Two supplementary doses were given 24 and 48 h after UV exposure (Fig. 1). In the long-term study, subjects received 100 mg Nutroxsun™, 250 mg Nutroxsun™, or the placebo product once a day at breakfast.

### Primary and secondary outcomes

The primary endpoints with respect to the photoprotective efficacy were the measurement of the UVB-induced skin redness, the assessment of the erythemal response of the skin after UVB exposure (290–320 nm), and the measurement of the basal and UVA-stimulated (320–400 nm) skin LPO content. The primary endpoint with respect to the antiageing efficacy was the measurement of the wrinkle depth. Skin elasticity was measured as a secondary efficacy endpoint. The study flow and the schedule of assessments chart are reported in Fig. 1.

### Measurement of skin redness

A spectrophotometer/colorimeter CM-700D (Konica Minolta, Milano, Italy) was used to measure skin redness in the CIELab colour space. The a* (red-green) parameter was measured in the UVB-exposed skin site to 1 MED. Measurements were taken excluding the specular reflection. The specular component excluded mode, provides results similar to those observed visually.

### Assessment of minimal erythemal dose

**Fig. 2:**
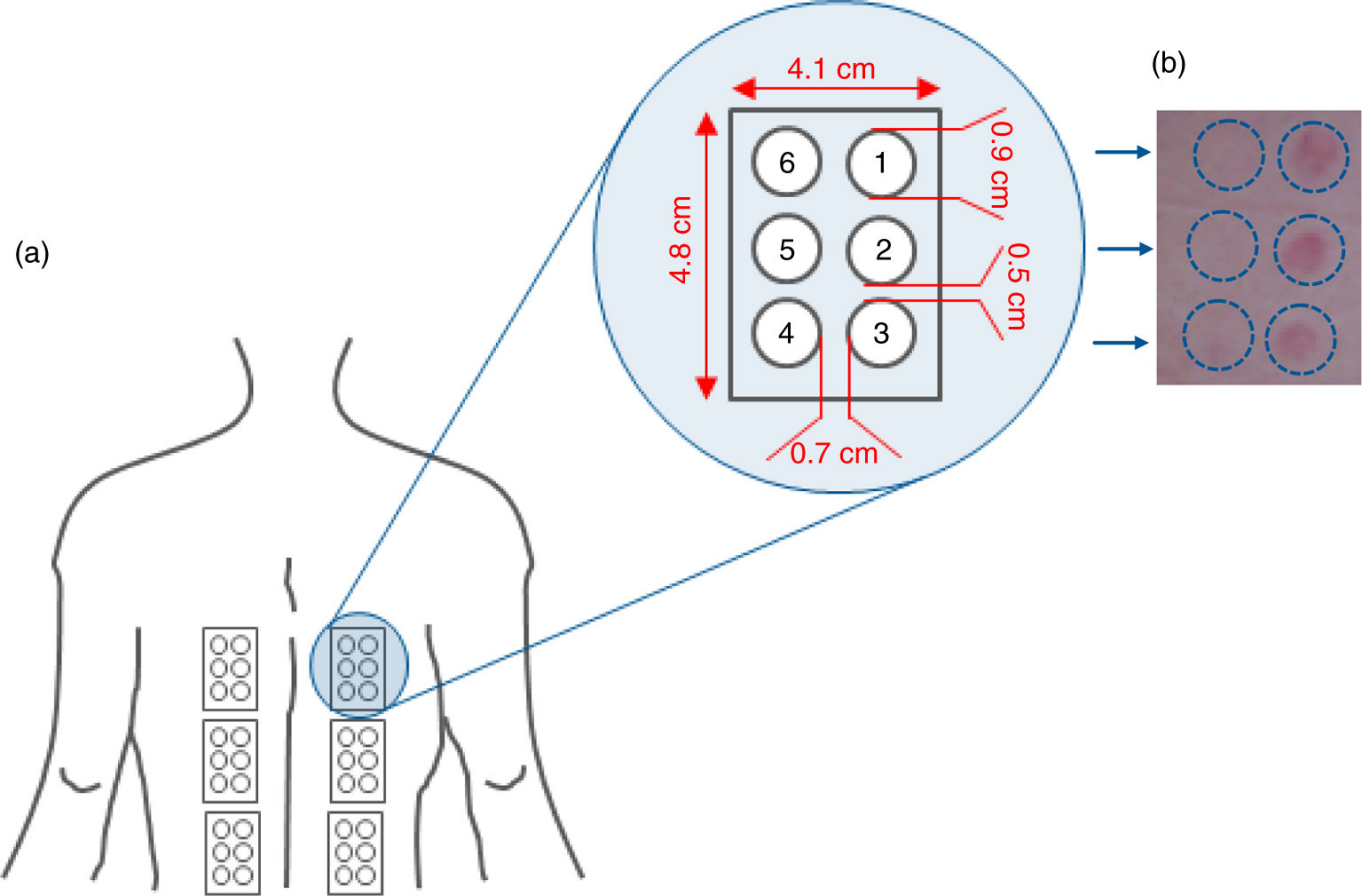
(a) UV exposure site and subsites. (b) Minimal erythema dose (MED).

One day before the study began, a provisional MED was determined in order to centre the UV doses ranges for the MED assessment through the study. A series of UVB doses (geometric progression of 1.25×), were applied on six small subsites (Fig. 2) of the skin of the back. MED was then assessed, under blind conditions, 20±4 h after UV exposure. MED assessment was carried out in a room with matt neutral wall colour and sufficient illumination conditions (at least 450 lux). MED assessment was considered invalid when: 1) the series of UVB exposures on a subject failed to elicit an erythemal response on any sub-site, 2) all subsites in the exposure series showed an erythemal response, and 3) erythemal responses within an exposure series were randomly absent. The source of UVB radiation was a Multiport 601–300 W Solar simulator (Solar^®^ Light Co. Inc., Philadelphia, USA) compliant with ISO 24444:2010 standard requirements (49). UVB dose was adjusted with a model PMA 2100 radiometer (Solar^®^ Light Co. Inc., Philadelphia, USA) equipped with a PMA 2103 LLG SUV detector (Solar^®^ Light Co. Inc., Philadelphia, USA). Both the solar simulator and the radiometers were calibrated externally.

### Assessment of LPO

Basal and UVA-induced (10 J/cm^2^) skin LPO were measured in the 10th skin layer obtained using the skin stripping technique. Skin stripping was performed in the back (Fig. 2) using Corneofix^®^ foils (Courage+Khazaka Electronic, Köln, Germany) under standard pressure conditions (225 g/cm^2^). The first stripping was discarded while strip no. 11 was collected and stored at −80°C until further analysis. Malondialdehyde (MDA) was measured according to the assay described by Erdelmeier et al. in 1998 (50) with minor modification, as follows: 1) skin strippings were layered in 12 multiwell plates containing 500 µl of a 0.5 mM CuSO_4_ aqueous solution, 2) multiwell plates were incubated at 37°C, using a microplate incubator/shaker under continuous agitation for 1 h, 3) after incubation 1.3 ml R1 solution (2.13 mg N-methyl-2-phenylindole/ml acetonitrile) and 0.3 ml 37% HCl was added and samples were further incubated at 45°C for 60 min under continuous agitation, 4) the reaction was stopped in ice for 10 min followed by 10 min at room temperature, 5) 1 ml of solution was centrifuged at 13,000 rpm per 10 min, and 6) absorbance was read at 586 nm using a multiwell plates reader (programmable MPT reader model DV 990BV6; Gio DeVita & C, Rome, Italy).

The source of UVA radiation was a Multiport 601–300 W Solar simulator (Solar^®^ Light Co. Inc., Philadelphia, USA) compliant with the Japan Cosmetic Industry Association (JCIA) measurement standard for UVA protection (51) and ISO 24442:2011 standard requirements (52) (Table 1). UVA dose was adjusted with a model PMA 2100 radiometer (Solar^®^ Light Co. Inc., Philadelphia, USA) equipped with a PMA 2113 LLG UVA detector (Solar^®^ Light Co. Inc., Philadelphia, USA). Both the solar simulator and the radiometers were calibrated and compliant to ISO 24444:2010 standard.

### Wrinkle depth

Wrinkles depth was measured using a three-dimensional (3-D) microtopography imaging system (PRIMOS 3D lite, GFMesstechnik GmbH, Teltow, Germany). The imaging system projects structured light on a specific surface of the skin with a digital micro-mirror device (DMD, Texas Instruments, Irving, TX, USA) and records the image with a CCD camera. Skin surface microtopography is then reconstructed using temporal phase shift algorithms to generate 3-D images. The imaging system has an overlap feature which enables precise matching of photos taken at different visits. In order to improve image overlap, subjects’ position was regulated using a stereotactic device (Canfield Scientific, Inc., Fairfield, NJ, USA). Wrinkle depth was measured in the periocular area (‘crow's feet wrinkles’) using the appropriate software routine.

### Skin elasticity

**Fig. 3:**
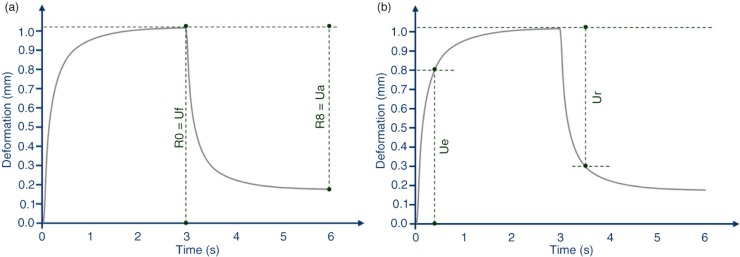
Skin elasticity curve. (a) R2 parameter calculation. (b) R5 parameter calculation.

A skin viscoelasticity analyser (Cutometer^®^ MPA 580, Courage+Khazaka Electronic, Köln, Germany) was used to measure skin elasticity. The skin surface of the face (cheek) was drawn into the aperture (3 mm) of the probe by a negative pressure (450 mbar) for 3 sec and thereafter released for 3 sec. The penetration depth of the skin inside the probe, during the suction and the release phase, was measured by a non-contact optical measuring system. Two skin elasticity indices were measured: 1) R2 (Ua/Uf, gross elasticity or overall elasticity, Fig. 3a represents the ability of redeformation of the skin to its basal state, and 2) R5 (Ur/Ue, net elasticity, Fig. 3b represents the elastic recovery of the skin deformation to its basal state due to its elastic component.

### Sample size

Sample size was calculated, for the long-term study, with a two-sided 5% significance level and a power of 80% taking into account a 20% variation of the primary endpoints due to both inter-individual human variability and error in the measurement techniques. Sample size was calculated using PASS 11 statistical software (version 11.0.8 for Windows) running on Windows Server 2008 R2 Standard SP1 64 bit edition (Microsoft, USA). A sample size of 20 subjects per group was necessary given an anticipated dropout rate of 20%.

### Randomisation

A restricted randomisation list was created using PASS 11 (version 11.0.8; PASS, LLC. Kaysville, UT, USA) statistical software running on Windows Server 2008 R2 Standard SP1 64 bit edition (Microsoft, USA) by a biostatistician and stored in a safe place. Randomisation sequence was stratified using 10% maximum allowable % deviation with a 1:1:1 allocation ratio. The allocation sequence was concealed from the study director in sequentially numbered, opaque, and sealed envelopes, reporting the unblinded treatment allocation (based on subject entry number in the study). The A4 sheet reporting the unblinded treatment was folded to render the envelope impermeable to intense light. After acceptance of the subject in the study the appropriate numbered envelope was opened. An independent technician dispensed either active or placebo products according to the card inside the envelope. The study adhered to established procedures to maintain separation between the investigator and its collaborators and the staff that delivered the intervention. Investigator and its collaborators who obtained outcome measurements were not informed on the product group assignment. Staff who delivered the intervention did not take outcome measurements. Subjects, investigator and collaborators were kept masked to products assignment. The active and the placebo products were in capsule form and identical in appearance. They were prepacked in blisters and consecutively numbered for each subject according to the randomisation schedule. Each subject was assigned an order number and received the capsules in the corresponding prepacked blister.

### Statistical methods

Statistical analysis was performed using NCSS 8 (version 8.0.4 for Windows; NCSS, Kaysville, UT, USA) running on Windows Server 2008 R2 Standard SP1 64 bit edition (Microsoft, USA). Data normality was checked using Shapiro–Wilk W normality test and data shape. Intragroup (vs. baseline) statistical analysis was carried out using repeated measures analysis of variance (RM-ANOVA) followed by Tukey–Kramer post-test. Intergroup (between treatments) statistical analysis was carried out using multivariate analysis of variance (M-ANOVA) followed by two-way t test of Student. A *p*<0.05 was considered statistically significant. Statistical analysis output was reported as follows: * *p*<0.05, ** *p*<0.01, and *** *p*<0.001.

## Results

### Subjects

**Fig. 4:**
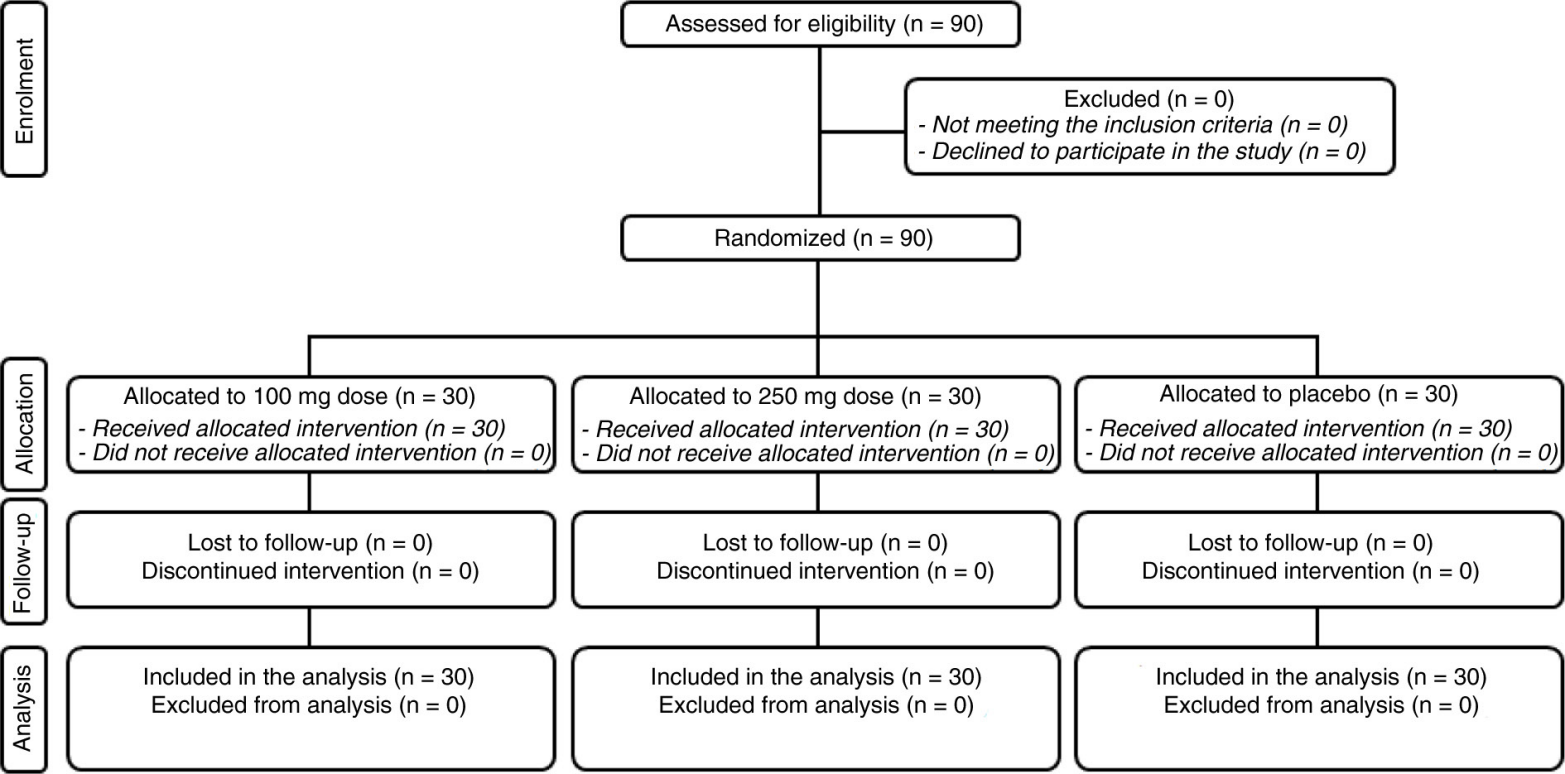
Flow chart of inclusion of subjects.

The study was conducted between February and April 2015. A total of 90 female subjects were successfully randomised (Fig. 4) in the long-term study while a total of five female subjects were enrolled in the short-term study. The population was Caucasian. Demographic and baseline characteristics (Table 2) were similar across treatment arms, indicating an unbiased randomisation and the absence of covariates. Subjects participating in the short-term pilot study attended clinic visits at the time of randomisation (baseline) and 24, 48 and 72 h after UVB exposure and the first product intake; while remained at our clinical facilities where the measure was done 1 and 4 h the day after UVB exposure. In the long-term study, subjects attended clinic visits at the time of randomisation (baseline) and after 14 days, 1, and 2 months of product use. Data analysis was intention-to-treat and involved all subjects who were randomly assigned. Subjects’ compliance to treatment was assessed by means of product accountability, as follows: at each visit, the expected amount of consumed capsule was compared with the amount dispensed minus the amount the subject returned. No major deviation was observed in the treatment regimen. All subjects were included in the safety analysis data set. All the tested products were well tolerated. No adverse reactions occurred during the study period.

**Table 1:** UVB and UVA solar simulator specifications

	% RCEE			
			
Spectral range (nm)	Lower limit	Upper limit	Spectral range (nm)	Limit (%)
<290	–	<0.1	UVA I (340–400 nm) irradiance	≥60
290–300	2.0	8.0	UVA II (320–340 nm) irradiance	≥20
290–310	49.0	65.0	**Total energy**	<150 mW/cm^2^
290–320	85.0	90.0	**UVA/Total**	92–100
290–330	91.5	95.5	**UVB/UVA**	<0.1
290–340	94.0	97.0	**UVA2/UVA**	8.0–20.0
290–350	95.5	98.5	**Spectrum continuity**	Continuous

**Table 2:** Demographic and baseline characteristics

	STT			LTT			
			
	100 mg	250 mg	Placebo	100 mg	250 mg	Placebo	Units
Sex							
Male	0	0	0	0	0	0	No.
Female	5	5	5	30	30	30	No.
Skin phototype							
I	–	–	–	6.7%	6.7%	6.7%	%
II	40%	40%	40%	33.3%	30.0%	36.7%	%
III	60%	60%	60%	60.0%	56.7%	50.0%	%
Age	30.8	30.8	30.8	52.9	51.0	50.9	years
Skin erythema (basal)	7.1	7.0	7.1	–	–	–	a.u.
Skin erythema (after UVB)	9.7	9.5	9.9	–	–	–	a.u.
Minimal erythema dose (MED)	–	–	–	29.0	30.3	29.3	mJ/cm^2^
LPO (basal)	–	–	–	2.61	2.72	2.58	µM MDA
LPO 4 h at D0	–	–	–	3.60	3.64	3.54	µM MDA
LPO 24 h at D0	–	–	–	3.23	3.30	3.21	µM MDA
Wrinkle depth	–	–	–	296.6	257.7	282.6	µm
Skin elasticity (R2 = Ua/Uf)	–	–	–	0.7233	0.7271	0.7214	Ratio
Skin elasticity (R5 = Ur/Ue)	–	–	–	0.2856	0.2940	0.2907	Ratio

Data are means±SE.

### Effect of the extracts on UVB-induced skin redness

**Fig. 5:**
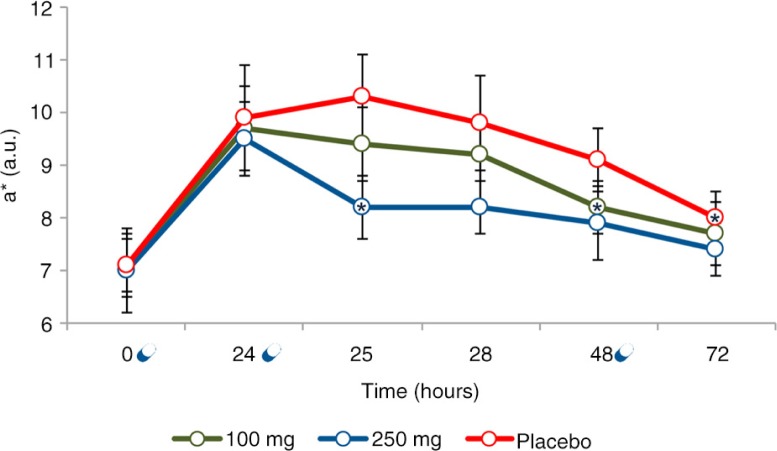
Skin redness time course after 1 MED UVB exposure. Data are means (arbitrary units)±SE. *Statistically significant (*p*<0.05) when compared to 24 h; 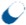 Product intake.

**Fig. 6:**
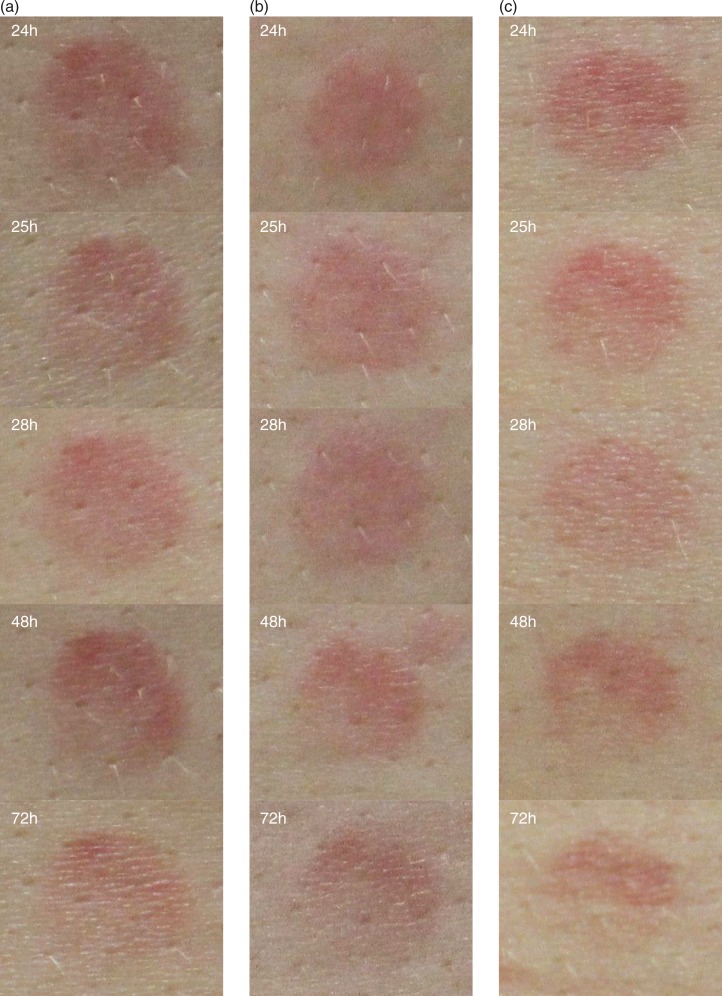
Skin redness variation after 1 MED UVB exposure. Digital pictures of (a) placebo, (b) 100 mg extracts dose, and (c) 250 mg extracts dose were taken using a Nikon D300 camera (Nikon corporate, Japan) equipped with a Nikon macro lens (AF-S Micro Nikkor 60 mm f/2.8 G ED) and parallel-polarised filters.

Twenty-four hours after UVB exposure to 1 MED, skin redness was increased by 40.5% (*p*=0.0099) in the placebo group, by 37.0% (*p*=0.0011) in the 100 mg dose group, and 39.6% (*p*=0.0006) in the 250 mg dose group (Fig. 5). The effect of UVB on skin redness was similar for all treatments (*p*=0.9387). Skin redness time course (variation vs. 24 h) exhibited a decrease compared to placebo as the extract dose was increased from 100 to 250 mg. A statistical significant variation was observed at 48 h in the 100 mg dose group (*p=*0.0252), and at 25 h (*p*=0.0437) in the 250 mg dose group, compared to 24 h data point. At 72 h for both the 100 and 250 mg dose groups skin redness returned to its basal (pre-UVB-exposure) value. In the placebo-treated group, skin redness showed a significant decrease at 72 h (*p*=0.0112), but even it remained slightly higher when compared to its basal value (*p*=0.0289). Despite the evident differences in time course behaviour of skin redness between 100 and 250 mg dose groups, this variation was not statistically significant (*p*=0.3720) when 100 and 250 mg data were compared (Fig. 6). The variation of skin redness observed for both 100 and 250 mg extracts dose treatment regimen was statistically significant when compared to placebo treatment regimen (100 mg group, *p*=0.020; 250 mg group, *p*=0.0182).

### Effects on MED

**Fig. 7:**
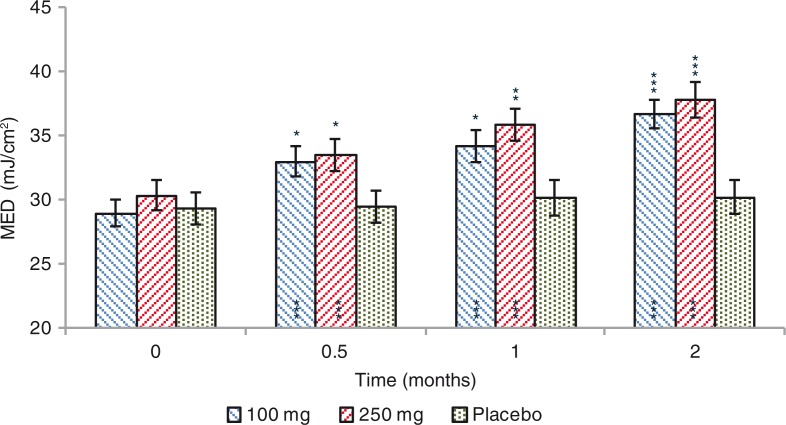
Minimal erythemal dose (MED) before and after 0.5, 1, and 2 months treatment. Intragroup (vs. 0) statistical analysis is reported inside the bars of the histogram. Intergroup (vs. placebo) statistical analysis is reported upon the bars of the histogram. Statistical analysis is reported as follows: *p < 0.05, **p < 0.01, and ***p < 0.001. Data are means (mJ/cm^2^)±SE.

**Fig. 8:**
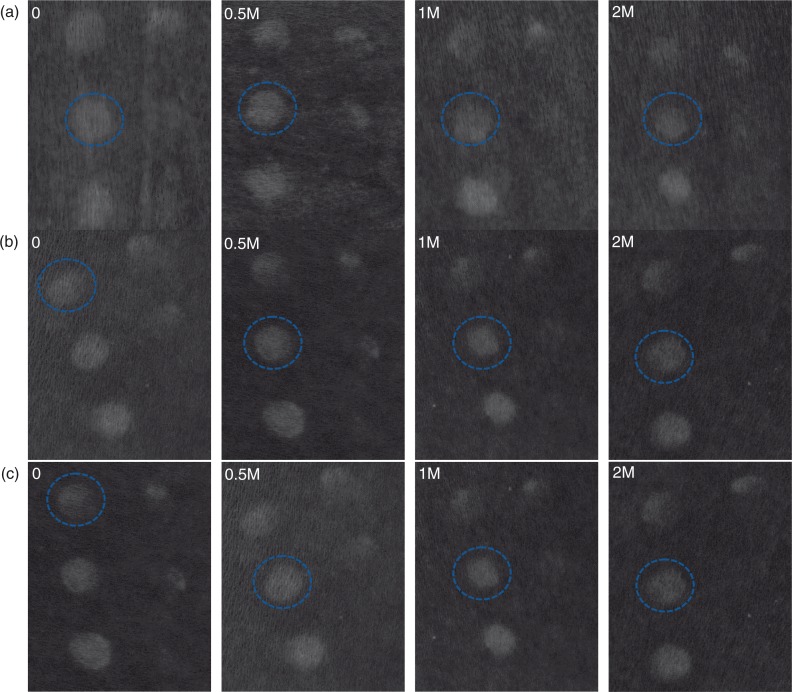
Digital pictures of (a) placebo, (b) 100 mg extracts dose, and (c) 250 mg extracts dose were taken using a Nikon D300 camera (Nikon corporate, Japan) equipped with a Nikon macro lens (AF-S Micro Nikkor 60 mm f/2.8 G ED) and parallel-polarised filters. The a* (CIELab chromatic space) channel image is reported in order to enhance image contrast. The blue circle indicates the MED.

A significant increase of the MED was observed for both 100 and 250 mg dose groups (Fig. 7). MED increased by 4.0 (+15.2%), 5.2 (+20.5%), and 7.7 (+29.8%) mJ/cm^2^, after 0.5, 1, and 2 months treatment, respectively (*p*=0.0000) in the 100 mg dose group. A similar efficacy profile was seen for the 250 mg dose group, where MED was increased by 3.1 (+11.7%), 5.5 (+20.2%), and 7.5 (+26.9%) mJ/cm^2^, after 0.5, 1, and 2 months treatment,respectively (*p*=0.0007 at 0.5 months and *p*=0.0000 at 1 and 2 months). Variation of MED was not statistically significant (*p*=0.1857) when 100 mg and 250 mg data were compared. MED variation observed for both 100 mg and 250 mg extract dose groups was statistically significant when compared to placebo group (100 mg group, *p*=0.0001; 250 mg group, *p*=0.0000). MED was unchanged (*p*=0.4049) in the placebo-treated subjects (Fig. 8).

### Horny layer lipoperoxides content

#### Basal lipid peroxidation

A significant decrease of the basal, not UVA stimulated, LPO content was observed for both 100 and 250 mg dose groups (Table 3). Skin horny layer MDA content was decreased by 14.4, 18.5 and 18.7% after 0.5, 1, and 2 months treatment, respectively (*p*=0.0000) in the 100 mg dose group. A bigger decrease (*p*=0.0038 compared to 100 mg) in the horny layer MDA content was observed for the 250 mg dose group, where MDA content was decreased by 25.5, 37.7, and 32.6% respectively (*p*=0.0000). MDA variation observed for both 100 and 250 mg dose groups was statistically significant when compared to placebo treatment regimen (*p*=0.0000). MDA was unchanged (*p*=0.1054) in the placebo-treated subjects.

**Table 3:** Basal and UVA-stimulated horny layer MDA content

Basal level	0		0.5 M		1 M		2 M	
100 mg	2.61±0.13		2.17±0.09*** (−14.4%)		2.01±0.07*** (−18.5%)		1.98±0.05*** (−18.7%)	
250 mg	2.72±0.16		1.94±0.12*** (−25.5%)		1.60±0.08*** (−37.7%)		1.69±0.07*** (−32.6%)	
Placebo	2.58±0.10		2.60±0.11 (+1.3%)		2.50±0.09 (−1.2%)		2.47±0.09 (−2.3%)	
UVA stimulated	0		0.5 M		1 M		2 M	
	4 h	24 h	4 h	24 h	4 h	24 h	4 h	24 h
100 mg	40.8%	25.6%	31.1%**	16.9%***	24.6%***	12.2%***	20.7%***	10.5%***
250 mg	37.5%	24.0%	27.3%***	14.9%***	21.1%***	10.7%***	15.8%***	8.2%***
Placebo	39.7%	26.3%	44.7%***	30.8%***	45.1%***	30.7%***	49.0%***	30.1%***

Data are means (µM MDA)±SE. Values in brackets: % variation vs. 0.

* *p <* 0.05

** *p <* 0.01

*** *p <* 0.001. UVA stimulated LPOs are % variation vs. baseline.

#### UVA-stimulated lipid peroxidation

A significant decrease of the UVA-stimulated LPO content was also observed, after 4 and 24 h from UVA exposure (10 J/cm^2^), for both 100 and 250 mg dose groups (Table 3) compared to baseline. Skin horny layer MDA content 4 h after UVA exposure was decreased by 9.7, 16.2 and 20.1% after 0.5, 1, and 2 months treatment, respectively (*p*=0.0000) in the 100 mg dose group while 24 h after UVA exposure the MDA content was decreased by 8.7, 13.4, and 15.1% after 0.5, 1, and 2 months treatment, respectively (*p*=0.0000). A similar efficacy profile was seen for the 250 mg dose group, where MDA, 4 h after UVA exposure, was decreased by 10.2, 16.4, and 21.7% after 0.5, 1, and 2 months treatment, respectively (*p*=0.0000); while 24 h after UVA exposure the MDA content was decreased by 9.1, 13.3, and 15.8% after 0.5, 1, and 2 months treatment, respectively (*p*=0.0000). MDA variation observed for both 100 and 250 mg dose groups was also statistically significant when compared to placebo treatment (Table 3). MDA was unchanged (*p*=0.7952 at 4 h, and *p*=0.7384 at 24 h) in the placebo-treated subjects.

### Wrinkle depth

**Fig. 9:**
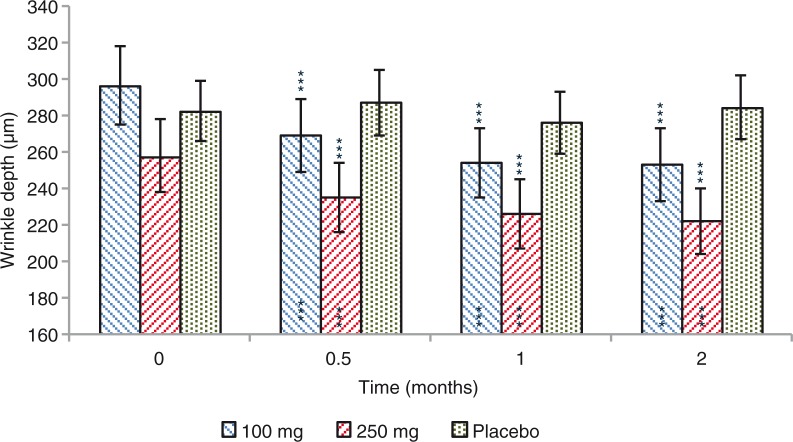
Wrinkle depth before and after 0.5, 1, and 2 months treatment. Intragroup (vs. 0) statistical analysis is reported inside the bars of the histogram. Intergroup (vs. placebo) statistical analysis is reported upon the bars of the histogram. Statistical analysis is reported as follows: *p < 0.05, **p < 0.01, and ***p < 0.001. Data are means (µm)±SE.

A significant decrease of the wrinkle depth in the ‘crow's feet’ was observed for both 100 and 250 mg dose groups (Fig. 9). Wrinkle depth decrease was by 27.4 (−8.8%), 42.4 (−13.4%), and 43.7 (−14.8%) µm, in the 100 mg dose group, after 0.5, 1, and 2 months treatment, respectively (*p*=0.0000). A similar efficacy profile was seen for the 250 mg extracts dose treatment regimen, where wrinkle depth was decreased by 22.7 (−9.1%), 31.5 (−12.6%), and 35.9 (−13.9%) µm, after 0.5, 1, and 2 months treatment, respectively (*p*=0.0001 at 0.5 months and *p*=0.0000 at 1 and 2 months). Variation of wrinkle depth was not statistically significant (*p*=0.7731) when 100 and 250 mg data were compared. Wrinkle depth variation observed for both 100 and 250 mg dose groups was statistically significant when compared to placebo treatment (*p*=0.0000). Wrinkle depth was unchanged (*p*=0.9740) in the placebo-treated subjects throughout the study.

### Skin elasticity

#### Gross elasticity (R2 parameter)

A significant increase of the skin gross elasticity (Fig. 3a) was observed for both 100 and 250 mg extracts dose treatment regimen (Table 4). Skin elasticity increased by 1.8, 3.2, and 4.6%, in the 100 mg dose group after 0.5, 1, and 2 months treatment, respectively (*p*=0.0026 at 0.5 months and *p*=0.0000 at 1 and 2 months). A similar efficacy profile was seen for the 250 mg dose group, where skin elasticity was increased by 1.5, 2.9, and 3.7%, after 0.5, 1, and 2 months treatment, respectively (*p*=0.0010 at 0.5 months; *p*=0.000 at 1 month and *p*=0.002 at 2 months). Variation of skin elasticity was not statistically significant (*p*=0.9253) when 100 and 250 mg were compared. Skin elasticity variation observed for both 100 and 250 mg dose groups was statistically significant when compared to placebo group (100 mg group, *p*=0.0455; 250 mg group, *p*=0.0281). Skin elasticity was unchanged (*p*=0.1355) in the placebo-treated subjects throughout the study.

**Table 4:** Skin elasticity

R2 (skin gross elasticity)	0	0.5 M	1 M	2 M
100 mg	0.7233±0.0135	0.7360±0.0134*** (+1.8%)	0.7462±0.0135*** (+3.2%)	0.7557±0.0136*** (+4.6%)
250 mg	0.7271±0.0127	0.7375±0.0129*** (+1.5%)	0.7475±0.0127*** (+2.9%)	0.7525±0.0113*** (+3.7%)
Placebo	0.7214±0.0114	0.7195±0.0116 (−0.3%)	0.7250±0.0120 (+0.5%)	0.7233±0.0121 (+0.2%)
R5 (skin net elasticity)	0	0.5 M	1 M	2 M
100 mg	0.2856±0.0111	0.2948±0.0115*** (+3.3%)	0.3020±0.0119*** (+5.8%)	0.3112±0.0121*** (+9.0%)
250 mg	0.2940±0.0098	0.3024±0.0101*** (+2.9%)	0.3093±0.0096*** (+5.5%)	0.3147±0.0098*** (+7.4%)
Placebo	0.2907±0.0091	0.2907±0.0096 (−0.1%)	0.2926±0.0089 (+0.8%)	0.2888±0.0088 (−0.5%)

Data are means ±SE. Values in brackets: % variation vs. 0.

*** *p <* 0.001.

#### Net elasticity (R5 parameter)

A significant increase of the skin net elasticity (Fig. 3b) was also observed for both 100 and 250 mg dose groups (Table 4). Skin elasticity increase was by 3.3, 5.8 and 9.0%, in the 100 mg dose group after 0.5, 1, and 2 months treatment, respectively (*p*=0.0000). A similar efficacy profile was seen for the 250 mg extracts dose treatment regimen, where skin elasticity was increased by 2.9, 5.5, and 7.4%, after 0.5, 1, and 2 months treatments, respectively (*p*=0.0000). Variation of skin elasticity was not statistically significant (*p*=0.2061) when compared 100 and 250 mg data were compared each other. Skin elasticity variation observed for both 100 and 250 mg extracts dose treatment regimen was statistically significant when compared to placebo group (*p*=0.0000). Skin elasticity was unchanged (*p*=0.2984) in the placebo-treated subjects throughout the study.

## Discussion

In recent years, different extracts derived from plants have been investigated for therapeutic application due to their pharmacological activity on inflammatory processes and other physiopathological conditions. Dietary interventions can interfere with several cell-signalling pathways and molecular targets may be involved in their efficacy in preventing or treating altered physiopathological conditions (53). Many plants, herbs and spices typically used for food flavouring and nutrition are excellent sources of phenolic compounds, which have been reported to show antioxidant activity. The anti-inflammatory activity of natural extracts has been associated to their antioxidant activity, and to a specific role on nitric oxide (NO) production suppression (54–56).

In a previous study, it was reported (46) the synergistic effects of a mixture of rosemary and citrus extracts in decreasing the generation of UVB-induced intracellular ROS and in preventing UVR-induced DNA damage in the comet assay. The mixture also showed genoprotective and antimutagenic properties in a model for massive generation of radical species using ionising radiation. A pilot trial in humans also showed the preliminary effect of the combination in increasing MED. Therefore, our present study aimed to investigate the anti-inflammatory, photoprotective, and antiageing effects of this combination. Two doses of the combination (100 and 250 mg) were investigated in order to assess if a dose-effect relationship between the measured parameters and the product intake exists.

The *in vivo* anti-inflammatory effect of the extracts on UVB-induced skin inflammation was investigated in a pilot (n=5 subjects) crossover study. Both 100 and 250 mg combination doses proved to be effective in decreasing the skin redness induced by 1 MED UVB exposure. As observed in the skin redness time course curves, the group having 250 mg dose of the combination recovered basal level in a much faster manner than that of the 100 mg dose group, clearly revealing a dose-dependent anti-inflammatory effect. The results also indicate the potential effect of the tested products in decreasing the UVB-induced skin redness with only 2 days of product consumption; however the small sample size and the associated high standard deviation was a limitation of the study and further studies would be required. Anyway, the results obtained in the pilot study provided the basis for sample size calculation.

In most studies on photoprotection based on nutritional ingredients, there is a time frame of approximately 6–10 weeks until protection against erythema becomes significant (57). A time frame much longer than we have seen in this study where the individual susceptibility to UVB radiation exposure (erythema) was decreased (+15.2 and +11.7% for 100 and 250 mg dose group, respectively) after 2 weeks of product use (Fig. 7). Two months after product use, the lowest dose of UVB radiation to produce the erythematous reaction was increased by about 7 mJ/cm^2^, corresponding to an increase around 33% of the time of sun exposure without experiencing sunburn. A result similar to that was obtained in a previous study where volunteers showed a 37% increase in the MED after 8 weeks of product use (46).

In the previous cell study using the same extract combination, part of the protective effect of rosemary and citrus polyphenols was assigned to their capacity of absorption/scattering of UVB radiation. However, this factor may have a negligible contribution *in vivo* due to the low concentration of polyphenols’ metabolites in skin cells.

The antioxidant properties of the skin metabolites derived from the compounds of the extracts combination may have a significant contribution to the observed UVR protective effects but further effects are expected to take place. The terpenes and caffeic acid derivatives from rosemary and citrus flavanones and flavones of the combination showed the capacity to scavenge first stage intracellular free radicals induced by UVR and ionising radiations such as such as superoxide radical anions (O_2_^•–^), H_2_O_2_, and hydroxyl radicals (OH^•^) (46, 58, 59). Furtherly, some of these radicals generate second stage lipoperoxy radicals (R–OO^•^) which are responsible for the generation of inflammatory mediators and generate DNA damage and protein oxidation. ROS are also considered inflammatory mediators through the activation of the NF-κB signalling, which controls the expression of pro-inflammatory cytokines. Therefore, the clinically visible increase of MED and the decrease of UVB-induced skin redness of the ingredient is not only due to their antioxidant capacity but also to their ability to attenuate the subsequent inflammatory response.

Moreover, it has been proven that some of these compounds are capable to reach intracellular targets and modulate multiple metabolic processes that go beyond their antioxidant properties (60). Hence, the polyphenols in the combination may be able to exert a direct modulation of the NF-κB signalling regardless their antioxidant capacity. In fact, rosemary polyphenols were shown *in vivo* to reduce the expression of several inflammation-associated genes which are regulated by NF-κB such as IL-1β, TNF-α, COX-1 and COX-2 in a mouse inflamed skin model, (61). In a keratinocyte HaCaT cell model stimulated with sodium lauryl sulphate, rosemary diterpenes also blocked the translocation of nuclear factor NF-κB by directly inhibiting its upstream signalling including (spleen tyrosine kinase) Syk/Src, phosphoinositide 3-kinase (PI3K) and protein kinase B (Akt) tyrosine kinases (62).

Skin LPO basal content (Table 3) was decreased indicating an effect of the extracts in improving the skin antioxidant status. Interestingly, the skin ability to counteract UVA-induced lipoperoxidation was also increased starting from 2 weeks of product use. Two months after product use, the UVA-induced LPO content was decreased by about 20 and 15%, 4 and 24 h after UVA exposure (Table 3). These results indicate that the metabolites derived from the ingredient are able to decrease the level of lipid peroxidation in the skin cells in only 2 weeks of consumption and therefore diminish the levels of skin LPO (lipoperoxy radicals, and MDA and hydroxynonenal as final products), which have been demonstrated to induce DNA and protein oxidation (63). Since LPO are also considered as inflammatory response mediators, their drop is also consistent with the observed decrease in skin redness after 2 weeks of product consumption.

In the present study, an improvement of the wrinkle depth (Fig. 9) and skin elasticity (Table 4) was also observed starting from 2 weeks of product use. No differences in the measured outcomes were found between 100 and 250 mg extracts dose regimen, indicating a *plateau* effect starting from the lowest dose.

This result reveals an improvement of the extracellular matrix status that is composed of proteoglycans, polysaccharides and proteins, which are responsible for skin elasticity and stiffness. UVR-induced ROS and inflammatory mediators have been shown to induce the activation of nuclear transcription complex AP-1, through intracellular kinases signalling activation (MAP kinases, p38 and JNK), leading to metalloproteinases (MMPs) activation and decreased expression of collagen and other matrix proteins with the final consequence of reduced dermal matrix formation (64). Therefore, the combined antioxidant and anti-inflammatory effects of the *in vivo* product metabolites together with their direct action on intracellular signalling pathways may be the responsible factors for the decreased signs of photoaged skin.

As the study protocol was implemented for all ages, skin phototype from I to III (the most susceptible to UVR), chrono- and photoaged skin, study results can be extended to the general population. The female gender selection does not represent a limitation for study results extending to the general population since the molecular, cellular, and tissue-specific events leading to inflammation, chrono- or photoageing are shared among genders.

## Conclusions

Our results confirm the previous *in vitro* and *in vivo* results (46) indicating a photoprotective and antiageing efficacy of a combination of two plants extracts obtained from dried rosemary (*R. officinalis*) leaves and grapefruits (*C. paradisi*). Long-term oral extract supplementation can contribute to skin protection by maintaining a steady-state systemic concentration of compounds capable of protecting the skin cells from UVR-induced alteration. Positive effects such as reduced UVR-induced erythema, decreased skin LPO, decreased wrinkle depth, and increased elasticity are noted as short as 2 weeks of product consumption. The putative mechanism for these effects is most probably to take place through the inhibition of UVR-induced ROS and the concomitant inflammatory markers (LPO and cytokines) together with their direct action on intracellular signalling pathways which are responsible for extracellular matrix degradation. In conclusion, the intake of Nutroxsun™ can be considered a complementary nutrition strategy to avoid the negative effects of sun exposure and photoageing. To the best of our knowledge, this is the first study demonstrating the antioxidant, photoprotective, and antiageing efficacy of this combination of plants extracts.
